# Drug-drug interactions between antithrombotics and direct-acting antivirals in hepatitis C virus (HCV) patients: A brief, updated report

**DOI:** 10.3389/fphar.2022.916361

**Published:** 2022-08-09

**Authors:** Mario Enrico Canonico, Giuseppe Damiano Sanna, Roberta Siciliano, Fernando Scudiero, Giovanni Esposito, Guido Parodi

**Affiliations:** ^1^ Department of Advanced Biomedical Sciences, Federico II University, Naples, Italy; ^2^ Clinical and Interventional Cardiology, Sassari University Hospital, Sassari, Italy; ^3^ Department of Cardiac Surgery, IRCCS Policlinico San Donato, San Donato Milanese, Italy; ^4^ Department of Cardiology, ASST Bergamo Est, Seriate, Italy; ^5^ Department of Medical, Surgical and Experimental Sciences, University of Sassari, Sassari, Italy; ^6^ Department of Cardiology, Ospedali Del Tigullio, Lavagna, Italy

**Keywords:** drug-drug interactions, hepatitis C virus, antiplatelet agents, anticoagulant agents, thrombo-haemorrhagic risk

## Abstract

Hepatitis C virus (HCV) is one of the leading causes of chronic liver disease affecting over 71 million people worldwide. An increased incidence of atherothrombotic events [e.g. coronary artery disease (CAD), atrial fibrillation (AF)] has been observed in HCV seropositive patients. On the other hand, an increased bleeding risk is another clinical issue, particularly in subjects with liver cirrhosis, gastroesophageal varices, portal hypertension, thrombocytopenia and alcohol consumption. The introduction and progressively greater use of direct-acting antivirals (DAAs) (instead of protease and polymerase inhibitors) during the last decade has enabled a sustained virological response to be achieved in a significant percentage of patients. However, due to the high cardiovascular risk profile in HCV-infected patients, the concomitant use of antithrombotic therapies is often required, bearing in mind the possible contraindications. For example, despite better pharmacokinetic and pharmacodynamic properties compared with vitamin K-antagonists, plasma level fluctuations of direct oral anticoagulants (DOACs) due to pathological conditions (e.g. chronic kidney diseases or hepatic cirrhosis) or drug-drug interactions (DDIs) may be of great importance as regards their safety profile and overall clinical benefit. We aimed to examine and briefly summarize the significant DDIs observed between antithrombotic and HCV antiviral drugs.

## Introduction

**FIGURE 1:**
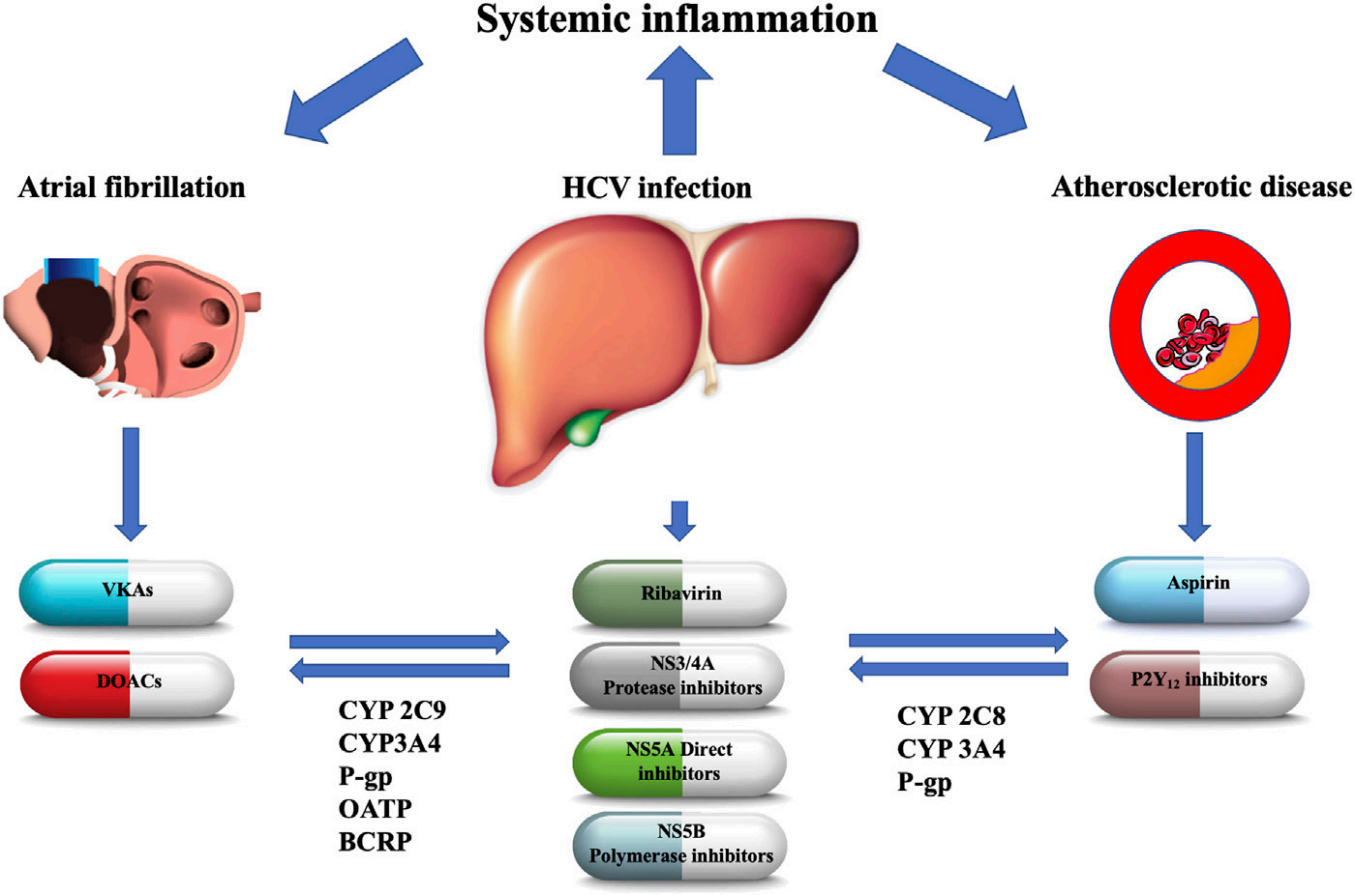
The main metabolic pathways of antiviral agents and oral antithrombotic drugs with their potential interactions. NS, nonstructural; CYP, cytochrome P450; P-gp, P-glycoprotein; BCRP, breast cancer resistance protein; OATP, organic anion-transporting polypeptide; VKAs, vitamin-K antagonists; DOACs, direct oral anticoagulants.

Hepatitis C virus (HCV) is one of the leading causes of chronic liver disease affecting several million people worldwide (Lee et al., 2019). The viral infection impacts on the liver by triggering metabolic derangements which may lead to steatosis, cirrhosis, cancer and death (Lee et al., 2019). Furthermore, HCV is associated with systemic inflammatory reactions, especially in the cardiometabolic risk factor setting (Lee et al., 2019). The relationships between HCV infection, chronic liver disease and atherosclerosis have not yet been completely elucidated. It has been hypothesized that HCV promotes atherogenesis and its complications through several mechanisms, including viral colonization within the arterial wall, liver steatosis and fibrosis, oxidative stress and enhanced production of inflammatory cytokines (Lee et al., 2019). An increased incidence of coronary artery disease (CAD) events has been observed in HCV seropositive patients, being higher in patients with detectable HCV ribonucleic acid (RNA) than in those with a remote infection (Scudiero et al., 2020). Moreover, as with other chronic diseases (e.g. obesity), patients with acute coronary syndromes (ACS) and HCV infection have increased platelet reactivity compared with those with no viral infection (Scudiero et al., 2020; Scudiero et al., 2022). HCV was confirmed to be an independent predictor of major adverse cardiovascular events (MACE) (Scudiero et al., 2020; Scudiero et al., 2022). In addition, patients with chronic HCV infection have a significantly higher risk of incidental atrial fibrillation (AF) and a higher prevalence of comorbid diseases (Yang et al., 2019). HCV patients have a higher baseline CHA_2_DS_2_‐VASc score, namely a clinical risk factor-based score including several variables [Congestive heart failure, Hypertension, Age ≥75 years, Diabetes mellitus, Stroke, Vascular disease, Age 65–74 years, Sex category (female)] (Yang et al., 2019). The link between HCV infection and AF is probably systemic inflammation (Yang et al., 2019), with possible additional risk factors not included in the CHA_2_DS_2_-VASc score. On the other hand, HCV patients with impaired liver function show an increased HAS-BLED score, a risk score to estimate the 1-year risk for major bleeding in AF patients—mainly those with liver cirrhosis and alcohol consumption - which once again includes several clinical risk factors [uncontrolled Hypertension, Abnormal renal and/or hepatic function, Stroke, Bleeding history or predisposition, Labile INR, Elderly, Drugs or excessive consumption of alcohol] (Lee et al., 2019). Bleeding risk associated with liver cirrhosis is related to gastroesophageal varices, portal hypertension and thrombocytopenia due to increased platelet destruction, and splenic and hepatic sequestration (Lee et al., 2019). Increased platelet destruction mechanisms include platelet autoantibodies and sequestration due to hypersplenism, while virus-induced bone marrow suppression and decreased thrombopoietin production are the main causes of reduced platelet production (Lee et al., 2019; Chang et al., 2021). Moreover, reduced levels of factors II, IX, XI, and XII are typical of liver cirrhosis and are associated with increased bleeding risk (Scudiero et al., 2020). Antithrombotic drugs increase the risk of bleeding especially in patients with concomitant liver disease (Chang et al., 2021). Of note, patients with moderate-severe liver disease (Child-Pugh class B-C) may benefit from further risk-benefit assessment by laboratory assay (liver function test, platelet count and coagulation profile), screening for alcohol consumption and concomitant drug prescription (Chang et al., 2021). Finally, the American Association for the Study of Liver Diseases also recommends screening for esophageal varices before beginning anticoagulants in these patients (Chang et al., 2021). The introduction over the last decade of DAAs contributed to radically changing the natural history of hepatitis C. Their use is associated with an undetectable HCV RNA level 12 weeks after treatment completion in at least 95% of patients, resulting in the prevention of HCV-related complications including the risk of MACE (Lee et al., 2019; Scudiero et al., 2020). However, due to the high cardiovascular risk profile in HCV-infected patients (including those with liver cancer), the use of antithrombotic therapies is required especially in cases of CAD and/or AF, always bearing in mind the concomitant bleeding risk. Careful thrombo-hemorrhagic assessment is needed in order to minimize the risk of adverse events related to pharmacological treatments (Scudiero et al., 2020; Santoro et al., 2021). DOACs show better pharmacokinetic and pharmacodynamic properties compared with vitamin K-antagonists (VKAs); however, plasma level fluctuations of this class of drug are common due to comorbidities (e.g. chronic kidney disease or hepatic cirrhosis) or drug-drug interactions (DDIs) which may be of great importance as regards their safety profile and overall clinical benefit (Gelosa et al., 2018). The most important drug-metabolizing enzymes involved in DDIs are usually cytochrome (CYP) P-450 and influx and efflux drug transporters such as P-glycoprotein (P-gp). Due to strong drug-inhibitors or inducers, the plasma concentration of metabolized drugs can be altered (Gelosa et al., 2018). Finally, moderate to severe liver disease impairs hepatic clearance of oral antithrombotic agents, increasing the already baseline high bleeding risk in these patients due to greater drug exposure (Chang et al., 2021). Moreover, the European Heart Rhythm Association Guidelines have contraindicated DOAC prescription in patients with severe liver disease (Child-Pugh class C) (Chang et al., 2021). DDIs constitute a clinical issue, especially in elderly patients with comorbidities. The direct relationship between advanced age, frailty, comorbidities and polypharmacotherapy leads to more frequent DDIs and related adverse events (Liu et al., 2018). In addition, DDI is a common finding in hemodialysis patients due to end-stage renal disease (ESRD). A recent study showed that HCV-viremic patients on hemodialysis had a very high prevalence of comedications with a subsequent risk for DDIs involving DAAs and other medications (including CV drugs) in a range between 6 and 25% (Hsu et al., 2021). We aimed to examine and briefly summarize the significant DDIs observed between antithrombotic and HCV antiviral drugs. Currently available antiviral drug classes were involved in DDI assessment: ribavirin and DAAs such as nonstructural protein (NS) 3/4A protease inhibitors, NS5A direct inhibitors and NS5B polymerase inhibitors. A search was conducted through the web-based engine PubMed in order to identify all peer-reviewed studies relevant to the topic published within the last 5 years (2016–2021). In addition, information from the Summary of Product Characteristics approved by the European Medicines Agency (EMA) and the United States Food and Drug Administration (FDA) regulatory authorities were used. Finally, the Liverpool Drug Interaction Database (http://www.hep-druginteractions.org) was used to identify additional DDIs. The main metabolic pathways of antiviral agents and antithrombotic drugs with their potential interactions are shown in Figure 1.

### Antiplatelet agents

Aspirin (acetylsalicylic acid) is transformed into salicylate in the stomach, intestinal mucosa, the blood and the liver (Nagelschmitz et al., 2014). To date, there are neither proven nor expected interactions with ribavirin or DAAs.

Among P2Y12 receptor inhibitors clopidogrel, a strong CYP2C8 inhibitor, markedly increased dasabuvir exposure and inhibited the CYP2C8-mediated formation of the primary M1 metabolite of dasabuvir (Itkonen et al., 2019). On the other hand, ritonavir, a strong CYP3A4 inhibitor, decreased plasma concentrations of the active metabolite of clopidogrel (Itkonen et al., 2019). In conclusion, clopidogrel markedly elevates dasabuvir concentrations, while patients receiving ritonavir are at risk for diminished clopidogrel response (Itkonen et al., 2019). Dasabuvir is frequently co-administered with ritonavir in the antiviral treatment regimen; thus, a probable reduction in clopidogrel active metabolite is expected in the case of regimens containing dasabuvir-ritonavir, such as the 3D protocol (Itkonen et al., 2019).

Prasugrel is also a prodrug that is activated to one metabolite (R‐138727) and numerous inactive metabolites by CYP3A4 and CYP2B6 (EMA. Efient: Summary of product characteristics, 2009). There are no expected interactions with ribavirin, direct inhibitors or sofosbuvir. The serum concentration of the active metabolite of prasugrel may be reduced when in combination with Telaprevir due to CYP3A4 inhibition (Kiang et al., 2013). Ticagrelor is not a prodrug and does not require metabolic action to perform antiplatelet activity (Zhou et al., 2011). Strong CYP3A4 inhibitors increase exposure to ticagrelor (due to reduced clearance) and their combined use is not recommended due to bleeding risk, while moderate CYP3A4 inhibitors have a mild effect on ticagrelor exposure and are not contraindicated. Conversely, potent CYP3A4 inducers may decrease exposure to ticagrelor (due to enhanced clearance) and hence reduce its efficacy. Ticagrelor is a weak inhibitor of CYP3A4 and could increase the exposure of drugs with a narrow therapeutic range (Zhou et al., 2011). There are no reported DDIs between ribavirin and ticagrelor. Serum concentration of ticagrelor can be increased when it is combined with paritaprevir, simeprevir, telaprevir and boceprevir by cytochrome P-450 CYP3A inhibition (Zhou et al., 2011). Due to the potential hazards of DDIs, concurrent administration of these drugs is contraindicated. Serum concentration of ledipasvir, ombitasvir, elbasvir, daclatasvir and simeprevir may be increased when combined with ticagrelor and these drugs should not be used in combination (Zhou et al., 2011). The orodispersible formulation of ticagrelor (ODT) has recently been tested in patients with ACS (Parodi et al., 2021). Although ODT could theoretically be absorbed in a large amount by the upper gastrointestinal tract, thus avoiding first-pass metabolism by the liver, the potential beneficial effects in terms of DDI reduction have not been investigated so far (Parodi et al., 2021). As a general rule, the administration of orodispersible tablets might be an alternative way through mucosal membrane absorption which minimizes first pass metabolism by the liver and, by consequence, the potential DDIs related to the CYP pathway (Parodi et al., 2021). Cangrelor, a non-thienopyridine adenosine triphosphate analogue, is a potent intravenous, rapid-acting, reversible P2Y12 receptor antagonist that inhibits platelet activation and aggregation (Kengreal (cangrelor) [prescribing information], 2015). Cangrelor plays a role in the activation of CYP2C9 and CYP3A4, but the systemic concentration cannot be considered clinically relevant. There is no evidence of CYP-relevant inhibition or induction by cangrelor or its metabolites at clinical concentrations, indicating that cangrelor does not interfere with the CYP metabolism of other concomitantly administered drugs (Kengreal (cangrelor) [prescribing information], 2015).

There are no data on DDIs between inhibitors of glycoprotein IIb/IIIa and the antiviral agents used in HCV (Table 1).

**TABLE 1:** Potential drug-drug interactions between HCV antiviral treatments and antithrombotic agents. In green: safe coadministration with no expected interactions based on current evidence. In red: unsafe and harmful coadministration based on strong evidence available. In yellow: careful clinical and lab (e.g. INR, hemoglobin, platelets, D-dimer) monitoring required due to less evidence available.

Antithrombotic agent	Mechanism of drug-to-drug interaction (DDI)	Ribavirin	NS3/4A protease inhibitors (boceprevir, glecaprevir, grazoprevir, paritaprevir, Simeprevir, ritonavir, telaprevir, voxilaprevir)	NS5A direct inhibitors (daclatasvir, elbasvir, ledipasvir, ombitasvir, pibrentasvir, Velpatasvir)	NS5B polymerase inhibitors (dasabuvir, sofosbuvir)
*Aspirin*		No expected interaction	No expected interaction	No expected interaction	No expected interaction
*Clopidogrel*	CYP2C8 inhibition	No expected interaction	Probable reduction of Clopidogrel active metabolite	No expected interaction	Clopidogrel elevates Dasabuvir concentrations
*Prasugrel*	CYP3A4 Inhibition	No expected interaction	Probable increased Prasugrel concentration	No expected interaction	No expected interaction
*Ticagrelor*	P-gp and CYP3A4 inhibition	No data yet.	Contraindicated. Increased Ticagrelor concentration	Probable increased Ticagrelor concentration	Probable increased Ticagrelor concentration
*Abciximab, Tirofiban, Eptifibatide*	No data yet.	No data yet.	No data yet.	No data yet.	No data yet.
*Cangrelor*	No data yet.	No data yet.	No data yet.	No data yet.	No data yet.
*VKAs*	CYP2C9 induction	Decreased Warfarin exposure	Decreased Warfarin exposure	Decreased Warfarin exposure	
				No expected interaction	No expected interaction
*UFH and LMWH*		No expected interaction	No expected interaction	No expected interaction	No expected interaction
*Fondaparinux*		No expected interaction	No expected interaction	No expected interaction	No expected interaction
*Apixaban*	P-gp, CYP3A4, OATP and/or BCRP inhibition	No expected interaction	Contraindicated. Increased Apixaban concentration when co-administered		
			Increased Apixaban exposure	Increased Apixaban exposure	No expected interaction
*Dabigatran*	P-gp, OATP and/or BRCP inhibition	No expected interaction	Contraindicated. Increased Dabigatran concentration when co-administered		No expected interaction
			Increased Dabigatran exposure	Increased Dabigatran exposure	
				Contraindicated. Increased Dabigatran concentration when co-administered	
*Edoxaban*	P-gp, OATP and/or BCRP inhibition	No expected interaction	Increased Edoxaban exposure	Contraindicated. Increased Edoxaban concentration when co-administered	
				Increased Edoxaban exposure	No expected interaction
*Rivaroxaban*	P-gp, CYP 3A4, OATP and/or BCRP inhibition	No expected interaction	Contraindicated. Increased Rivaroxaban concentration when co-administered		
			Increased Rivaroxaban exposure	Increased Rivaroxaban exposure	No expected interaction

NS, nonstructural; CYP, cytochrome P450; P-gp, P-glycoprotein; BCRP, breast cancer resistance protein; OATP, organic anion-transporting polypeptide; VKAs, Vitamin-K, antagonists; UFH, unfractioned heparin; LMWH, low molecular weight heparin.

### Anticoagulant agents

Warfarin is a racemic mixture of R- and S-enantiomer, in which S-enantiomer has 2–5 times more anticoagulant activity than R-enantiomer (Nagelschmitz et al., 2014). The major PD effect of this VKA is caused by the inhibition of factor II (thrombin). Warfarin is primarily metabolized by CYP2C9, and in part by CYP2C19 (Nagelschmitz et al., 2014). Ribavirin decreases warfarin exposure by CYP2C9 induction (De Carolis et al., 2016). Boceprevir, a protease inhibitor, induces CYP1A2 and CYP2C9 resulting in reduced concentrations of warfarin (De Carolis et al., 2016). Although there are no expected potentially fatal DDIs between warfarin and DAAs or polymerase inhibitors, close monitoring of International Normalized Ratio (INR) is needed in the case of concomitant administration of warfarin with elbasvir/grazoprevir or ledipasvir/sofosbuvir due to decreased warfarin exposure (De Carolis et al., 2016). Unfractionated heparin and low-molecular-weight heparins (LMWHs) exert their anticoagulant action by catalyzing the inactivation of thrombin by antithrombin III. The anticoagulant effect of these drugs is mediated by inactivation enzymes like thrombin, factor Xa (FX_a_) and IXa. They are not absorbed by oral administration and are typically administered by IV injection or the subcutaneous route. Pharmacokinetics are linear at recommended dosage rates with no expected interactions with CYP450 or P-gp systems (Hirsh et al., 2001). Fondaparinux inhibits thrombin generation via inactivation of factor Xa in a linear dose-dependent way. It is administered by the subcutaneous route with practically 100% bioavailability. No *in vitro* interaction with CYP450 or P-gp systems was found (Donat et al., 2002). DOACs have emerged as leading therapeutic alternatives with more effective, safe and convenient treatment options in thromboembolic settings (Nagelschmitz et al., 2014). Their pharmacodynamics depend on plasma bioavailability and any DDIs modifying plasma concentration may alter anticoagulant activity, thus increasing the probability of side effects (mainly bleeding events) (Foerster et al., 2020). Apixaban is an orally bioavailable, direct acting/reversible FXa inhibitor (Nagelschmitz et al., 2014). Oxidative metabolism of apixaban for the formation of all metabolites was predominantly catalyzed by CYP3A4/5, with minor contributions made by CYP1A2 and CYP2J2 (Bellesini et al., 2020). Apixaban has multiple elimination pathways. Most of the administered dose of apixaban (>50%) is detected in faeces and about 25% in urine (Bellesini et al., 2020). Strong inhibitors of both CYP3A4 and P-gp, like protease inhibitors (i.e. paritaprevir and ritonavir), can increase apixaban concentration with a subsequent risk of bleeding (Bellesini et al., 2020). DAAs can raise the serum concentration of apixaban due to CYP3A inhibition (i.e. daclatasvir). Other DAAs (velpatasvir and voxilaprevir) can increase apixaban concentration due to breast cancer resistance protein (BCRP), organic anion-transporting polypeptide (OATP) or P-gp inhibition (Bellesini et al., 2020). The metabolism of elbasvir and sofosbuvir may be decreased when combined with apixaban. Therefore, the use of apixaban is not recommended in patients with concomitant use of strong CYP3A4 and P-gp inhibitors. No dosage adjustment is required for apixaban when co-administered with less-potent inhibitors of CYP3A4, P-gp/OAPT (i.e. ledipasvir) (Bellesini et al., 2020). Dabigatran etexilate is rapidly converted by serine esterases into the active drug dabigatran. In contrast with warfarin, dabigatran is not a substrate, inducer or inhibitor of cytochrome P450 enzymes. Patients with moderate hepatic impairment do not show any consistent change when exposed to dabigatran. Dabigatran etexilate is a substrate of the P-gp, BCRP and organic OATP efflux transporter (Nagelschmitz et al., 2014). Increased serum concentrations of dabigatran are possible when it is co-administered with protease inhibitors (i.e. paritaprevir, ritonavir and simeprevir) due to CYP 3A4 and P-gp inhibition or DAAs, due to P-gp (i.e. paritaprevir, velpatasvir and voxilaprevir), OATP1B (i.e. ledipasvir, simeprevir, velpatasvir and voxilaprevir) and BCRP (i.e. elbasvir, velpatasvir and voxilaprevir) inhibition (Bellesini et al., 2020). Due to high bleeding risk dabigatran administration is contraindicated with sofosbuvir/velpatasvir/voxilaprevir or glecaprevir/pibrentasvir co-administration, and careful clinical monitoring is required with concomitant elbasvir/grazoprevir therapy due to P-gp inhibition (Bellesini et al., 2020). Edoxaban, a once-per-day non-vitamin K antagonist oral anticoagulant, is a direct, selective, reversible inhibitor of FXa (Nagelschmitz et al., 2014). Renal clearance accounts for approximately 50% of total clearance, and metabolism and biliary secretion for the remaining 50% (Nagelschmitz et al., 2014). Increased serum concentrations of edoxaban are expected when it is co-administered with P-gp inhibitors such as protease inhibitors (i.e. paritaprevir, ritonavir and simeprevir) or DAAs (i.e. ledipasvir, elbasvir, velpatasvir and voxilaprevir) for OATB1/BCRP inhibitions. Edoxaban administration is contraindicated with sofosbuvir/velpatasvir/voxilaprevir (Bellesini et al., 2020).

Rivaroxaban is an oral, direct FXa inhibitor, metabolized and eliminated both via several cytochrome P450 enzymes (CYP3A4/5, CYP2J2) and CYP-independent mechanism; whereas non-CYP-mediated hydrolysis accounts for 14% of total rivaroxaban elimination (by P-gp, BCPR and OATP) (Itkonen et al., 2019). The resulting metabolites are eliminated both in faeces and urine. The inhibition of P-gp or OATPB1 mediated by Protease inhibitors (i.e. paritaprevir and simeprevir) or DAAs (ledipasvir, velpatasvir and voxilaprevir) could result in increased rivaroxaban exposure with the potential for bleeding events. Serum concentration of rivaroxaban may be increased by P-gp and BCRP inhibition by DAAs during co-administration (i.e. elbasvir, velpatasvir and voxilaprevir). Velpatasvir and voxilaprevir can also increase rivaroxaban levels due to OATP inhibitions (Bellesini et al., 2020). The co-administration of rivaroxaban with ombitasvir/paritaprevir/ritonavir and dasabuvir is contraindicated due to increased bleeding risk (Bellesini et al., 2020). Finally, no DDIs are expected between DOACs and ribavirin (Table 1) (Bellesini et al., 2020). DDIs with DOACs constitute a clinical issue since DAAs are usually administered in combination regimens. In the absence of a universally accepted consensus, it might be reasonable to recommend some practical management strategies. Since HCV treatment typically lasts from 8 to 24 weeks, a reasonable approach should be a temporary interruption of DOACs and a bridge with LMWH until the end of antiviral treatments due to the absence of documented DDIs. This should be a useful strategy especially in patients with concomitant tumors (hepatocellular carcinoma) or severe liver dysfunction (cirrhosis). However, since subcutaneous administration of LMWH for long periods is clearly uncomfortable and potentially unsafe for patients, serial laboratory quantitative assessment of plasma drug concentrations in patients treated with DOACs during HCV therapy might be an alternative strategy, though not supported by scientific evidence so far.

## Conclusion

HCV infection causes systemic inflammation which in turn promotes atherosclerosis and AF. Due to chronic hepatic damage, HCV-antiviral and antithrombotic drug clearance is significantly impaired, with increased bleeding risk especially when co-administered. Drug dose adjustment based on plasma concentration constitutes a grey zone as data are missing. DDIs are a challenge for clinicians because of the need to balance the concomitant high bleeding and thrombotic risk related to the progression of HCV chronic infection with its systemic complications.

## References

[B1] Bellesini M. Bianchin M. Corradi C. Donadini M. P. Raschi E. Squizzato A. (2020). Drug-drug interactions between direct oral anticoagulants and hepatitis C direct-acting antiviral agents: Looking for evidence through a systematic review. Clin. Drug Investig. 40 (11), 1001–1008. 10.1007/s40261-020-00962-y PMC7595962 32809123

[B2] Chang W. H. Mueller S. H. Tan Y. Y. Lai A. G. (2021). Antithrombotic therapy in patients with liver disease: Population-based insights on variations in prescribing trends, adherence, persistence and impact on stroke and bleeding. Lancet Reg. Health. Eur. 10, 100222. 10.1016/j.lanepe.2021.100222 34806071 PMC8589727

[B3] De Carolis D. D. Westanmo A. D. Chen Y. C. Boese A. L. Walquist M. A. Rector T. S. (2016). Evaluation of a potential interaction between new regimens to treat hepatitis C and warfarin. Ann. Pharmacother. 50 (11), 909–917. 10.1177/1060028016660325 27465881

[B4] Donat F. Duret J. P. Santoni A. Cariou R. Necciari J. Magnani H. (2002). The pharmacokinetics of fondaparinux sodium in healthy volunteers. Clin. Pharmacokinet. 41 (2), 1–9. 10.2165/00003088-200241002-00001 12383039

[B5] EMA. Efient: Summary of product characteristics. 2009. Available at: http://www.ema.europa.eu/docs/en_GB/document_library/EPAR_Product_Information/human/000984/WC500021971.pdf . Accessed 6 Mar 2018.

[B6] Foerster K. I. Hermann S. Mikus G. Haefeli W. E. (2020). Drug-drug interactions with direct oral anticoagulants. Clin. Pharmacokinet. 59 (8), 967–980. 10.1007/s40262-020-00879-x 32157630 PMC7403169

[B7] Gelosa P. Castiglioni L. Tenconi M. Baldessin L. Racagni G. Corsini A. (2018). Pharmacokinetic drug interactions of the non-vitamin K antagonist oral anticoagulants (NOACs). Pharmacol. Res. 135, 60–79. 10.1016/j.phrs.2018.07.016 30040996

[B8] Hirsh J. Warkentin T. E. Shaughnessy S. G. Anand S. S. Halperin J. L. Raschke R. (2001). Heparin and low-molecular-weight heparin: Mechanisms of action, pharmacokinetics, dosing, monitoring, efficacy, and safety. Chest 119 (1), 64S–94S. 10.1378/chest.119.1_suppl.64s 11157643

[B9] Hsu P. Y. Wei Y. J. Lee J. J. Niu S. W. Huang J. C. Hsu C. T. (2021). Comedications and potential drug-drug interactions with direct-acting antivirals in hepatitis C patients on hemodialysis. Clin. Mol. Hepatol. 27 (1), 186–196. 10.3350/cmh.2020.0180 33317251 PMC7820195

[B10] Itkonen M. K. Tornio A. Lapatto-Reiniluoto O. Neuvonen M. Neuvonen P. J. Niemi M. (2019). Clopidogrel increases dasabuvir exposure with or without ritonavir, and ritonavir inhibits the bioactivation of clopidogrel. Clin. Pharmacol. Ther. 105 (1), 219–228. 10.1002/cpt.1099 29696643 PMC6585621

[B11] Kengreal (cangrelor) [prescribing information] (2015). Parsippany, NJ: The Medicines Company.

[B12] Kiang T. K. Wilby K. J. Ensom M. H. (2013). Telaprevir: Clinical pharmacokinetics, pharmacodynamics, and drug-drug interactions. Clin. Pharmacokinet. 52 (7), 487–510. 10.1007/s40262-013-0053-x 23553423

[B13] Lee K. K. Stelzle D. Bing R. Anwar M. Strachan F. Bashir S. (2019). Global burden of atherosclerotic cardiovascular disease in people with hepatitis C virus infection: A systematic review, meta-analysis, and modelling study. Lancet. Gastroenterol. Hepatol. 4 (10), 794–804. 10.1016/S2468-1253(19)30227-4 31377134 PMC6734111

[B14] Liu C. H. Yu M. L. Peng C. Y. Hsieh T. Y. Huang Y. H. Su W. W. (2018). Comorbidities, concomitant medications and potential drug-drug interactions with interferon-free direct-acting antiviral agents in hepatitis C patients in Taiwan. Aliment. Pharmacol. Ther. 48 (11-12), 1290–1300. 10.1111/apt.15011 30362139

[B15] Nagelschmitz J. Blunck M. Kraetzschmar J. Ludwig M. Wensing G. Hohlfeld T. (2014). Pharmacokinetics and pharmacodynamics of acetylsalicylic acid after intravenous and oral administration to healthy volunteers. Clin. Pharmacol. 6, 51–59. 10.2147/CPAA.S47895 24672263 PMC3964022

[B16] Parodi G. Talanas G. Mura E. Canonico M. E. Siciliano R. Guarino S. (2021). Orodispersible ticagrelor in acute coronary syndromes: The TASTER study. J. Am. Coll. Cardiol. 78 (3), 292–294. 10.1016/j.jacc.2021.05.015 34266583

[B17] Santoro C. Capone V. Canonico M. E. Gargiulo G. Esposito R. Sanna G. D. (2021). Single, dual, and triple antithrombotic therapy in cancer patients with coronary artery disease: Searching for evidence and personalized approaches. Semin. Thromb. Hemost. 47 (8), 950–961. 10.1055/s-0041-1726298 34261150

[B18] Scudiero F. Canonico M. E. Sanna G. D. Dossi F. Silverio A. Galasso G. (2022). Dual antiplatelet therapy with 3^rd^ Generation P2Y_12_ inhibitors in STEMI patients: Impact of body mass index on loading dose-response. Cardiovasc. Drugs Ther. 17. 10.1007/s10557-022-07322-2 35175499

[B19] Scudiero F. Valenti R. Marcucci R. Sanna G. D. Gori A. M. Migliorini A. (2020). Platelet reactivity in hepatitis C virus-infected patients on dual antiplatelet therapy for acute coronary syndrome. J. Am. Heart Assoc. 9 (18), e016441. 10.1161/JAHA.120.016441 32885738 PMC7726996

[B20] Yang Y. H. Chiang H. J. Yip H. K. Chen K. J. Chiang J. Y. Lee M. S. (2019). Risk of new-onset atrial fibrillation among asian chronic hepatitis C virus carriers: A nationwide population-based cohort study. J. Am. Heart Assoc. 8 (22), e012914. 10.1161/JAHA.119.012914 31711382 PMC6915266

[B21] Zhou D. Andersson T. B. Grimm S. W. (2011). *In vitro* evaluation of potential drug-drug interactions with ticagrelor: Cytochrome P450 reaction phenotyping, inhibition, induction, and differential kinetics. Drug Metab. Dispos. 39 (4), 703–710. 10.1124/dmd.110.037143 21177984

